# Impact on mental health and wellbeing in Indigenous communities due to land loss resulting from industrial resource development: protocol for a systematic review

**DOI:** 10.1186/s13643-022-02014-2

**Published:** 2022-07-20

**Authors:** Nicole Burns, Janice Linton, Nathaniel J. Pollock, Laura Jane Brubacher, Nadia Green, Arn Keeling, Alex Latta, Jessica Martin, Jenny Rand, Melody E. Morton Ninomiya

**Affiliations:** 1 Health Sciences, Faculty of Science, Wilfrid Laurier University, 75 University Ave W., Waterloo, Ontario N2L 3C5 Canada; 2 Neil John Maclean Health Sciences Library, University of Manitoba, 66 Chancellors Cir, Winnipeg, Manitoba R3T 2N2 Canada; 3 School of Arctic and Subarctic Studies, Labrador Campus, Memorial University, P.O. Box 490, Station B, Happy Valley-Goose Bay, Newfoundland and Labrador A0P 1E0 Canada; 4 School of Population and Public Health, University of British Columbia, 2206 East Mall, Vancouver, British Columbia V6T 1Z3 Canada; 5 Faculty of Nursing, University of Alberta, 116 ST & 85 Ave, Edmonton, Alberta T6G 2R3 Canada; 6 Department of Geography, Memorial University, 230 Elizabeth Ave, St. John’s, Newfoundland A1C 5S7 Canada; 7 Global Studies, Department of Geography and Environmental Studies, Wilfrid Laurier University, 75 University Ave. W, Waterloo, Ontario N2L 3C5 Canada; 8 Indigenous Studies, Cornell University, Ithaca, NY 14853 USA; 9 School of Health and Human Performance, Dalhousie University, 6299 South St, Halifax, Nova Scotia B3H 4R2 Canada; 10 Health Sciences, Faculty of Science, Wilfrid Laurier University, 75 University Ave W, Waterloo, Ontario N2L 3C5 Canada

**Keywords:** Mental health, Resource extraction, Industrial development, Indigenous Peoples, Land dispossession, Systematic review

## Abstract

**Background:**

Indigenous Peoples are impacted by industrial resource development that takes place on, or near, their communities. Existing literature on impacts of industrial resource development on Indigenous Peoples primarily focus on physical health outcomes and rarely focus on the mental health impacts. To understand the full range of long-term and anticipated health impacts of industrial resource development on Indigenous communities, mental health impacts must be examined. It is well-established that there is a connection between the environment and Indigenous wellbeing, across interrelated dimensions of mental, physical, emotional, and spiritual health.

**Methods:**

This paper identifies how the Community Advisory Team and a team of Indigenous and settler scholars will conduct the review. The literature search will use the OVID interface to search Medline, Embase, PsycINFO, and Global Health databases. Non-indexed peer-reviewed journals related to Indigenous health or research will be scanned. Books and book chapters will be identified in the Scopus and PsycINFO databases. The grey literature search will also include Google and be limited to reports published by government, academic, and non-profit organizations. Reference lists of key publications will be checked for additional relevant publications, including theses, dissertations, reports, and other articles not retrieved in the online searches. Additional sources may be recommended by team members. Included documents will focus on Indigenous Peoples in North America, South America, Australia, Aotearoa New Zealand, and Circumpolar regions, research that reports on mental health, and research that is based on land loss connected to dams, mines, agriculture, or petroleum development. Literature that meets the inclusion criteria will be screened at the title/abstract and full-text stages by two team members in Covidence. The included literature will be rated with a quality appraisal tool and information will be extracted by two team members; a consensus of information will be reached and be submitted for analysis.

**Discussion:**

The synthesized evidence from this review is relevant for land use policy, health impact assessments, economic development, mental health service planning, and communities engaging in development projects.

**Systematic review registration:**

Registered in the International Prospective Register of Systematic Reviews (PROSPERO; Registration number CRD42021253720)

**Supplementary Information:**

The online version contains supplementary material available at 10.1186/s13643-022-02014-2.

## Background

Many industrial resource development projects take place in close proximity to Indigenous communities, and in some cases, on Indigenous treaty lands [1–5]. While some Indigenous communities have worked collaboratively with industries and governments to establish sustainable resource developments [6], others have experienced non-collaborative processes which have resulted in land dispossession through project construction or community relocation [5, 7]. Several studies of land dispossession have reported negative health outcomes for Indigenous Peoples [8–10]. Assessing the expected and potential health impacts of land loss through industrial resource developments can provide Indigenous communities, industries, and governments with evidence that is necessary for planning, negotiation, mitigation, and monitoring.

There is a substantial body of evidence about the occupational and community health consequences associated with industries such as mining, energy, and agriculture [7, 10–14]. However, two recent scoping reviews [7, 10] highlighted the comparative lack of evidence about the mental health impacts of industrial resource developments. Most studies have focused on physical health outcomes and industry workers, with limited investigations about mental health or with Indigenous Peoples [7, 10].

Understanding the possible impacts of industrial resource development on mental health in Indigenous communities requires a recognition of the connection between the environment and Indigenous health and wellbeing [15, 16]. The relationship between Indigenous Peoples and land is part of a holistic ontology that situates mental health as interconnected with all other dimensions of health (physical, emotional, and spiritual) and with cultural identity and place [16–21]. Given these links, industrial resource developments that have physical impacts on Indigenous lands and territories, change access to land-based activities, or result in community displacement may serve as a unique pathway for mental health risks and outcomes in Indigenous communities.

### Objective and question

The objective of this systematic review is to examine and synthesize the evidence related to the mental health impacts of industrial resource development on Indigenous communities, with a specific focus on consequences due to land loss. This review will examine the reported impacts of land dispossession due to mining, hydroelectric, petroleum, and agricultural developments on mental health in Indigenous communities.

## Methods

This protocol was registered in the International Prospective Register of Systematic Reviews (Registration number CRD42021253720) and has been prepared in accordance with the statement for Preferred Reporting Items for Systematic Review and Meta-Analysis Protocols (PRISMA-P) [22, 23]. The completed PRISMA-P Checklist is available as Additional file 1.

We will conduct a systematic review of the peer reviewed and grey literature on the impact of land loss related to industrial resource development on mental health and wellbeing in Indigenous communities. This protocol was developed by an interdisciplinary team of Indigenous (NG and JM) and settler (non-Indigenous) scholars (NB, JL, NP, LJB, AK, AL, JR, MMN) with expertise in Indigenous health and wellbeing (NG, JM, NB, NP, LJB, JR, MMN), environmental stewardship (AK, AL), mental health (NG, NP, MMN), resource extraction (AK, AL), and Indigenous People’s health literature databases (JL). Collectively, the co-authors have expertise in quantitative, qualitative, and mixed research methodologies. In keeping with principles and practices for community-based participatory [24] and Indigenous research [25–29], this review was designed with support from a Community Advisory Team, comprised of four Indigenous and one settler members. Their role was to advise on the research objective and question, the inclusion and exclusion criteria, and the information that will be extracted from eligible studies. Our approach is founded on the ethical imperative of “nothing about us without us” in Indigenous research [16, 26, 30–32]. In this case, this systematic review was requested by West Moberly First Nations so that the evidence can support mental health and wellness planning, decision-making, and programming.

### Search strategy

An academic health sciences librarian with over twenty years of experience working in Indigenous health has developed the search strategy. In consultation with team members, keywords and topics have been translated to the appropriate subject headings. Test searches have been carried out in Scopus and Medline to gather data that includes studies focused in the following three concept areas: (1) Indigenous Peoples in global regions with similar histories of colonization (North America, South America, Australia, Aotearoa New Zealand, and Circumpolar regions); (2) mental health risks and outcomes such as mental health and wellness, psychological trauma, substance use, resilience; and (3) land loss connected to specific resource development industries (hydroelectric dams, mining, agriculture, petroleum). Please refer to the detailed search strategies developed to search Medline and Scopus for a full list of keywords and medical subject headings (MeSH terms) relevant to these two databases (Additional file 2). The OVID interface will be used to search Medline, Embase, PsycINFO, and Global Health for research published in English, up to and including December 31, 2021.

Five peer-reviewed journals related to Indigenous health or research will be scanned or hand searched by members of the team to identify potentially relevant articles; International Journal of Indigenous Health, International Indigenous Policy Journal, Journal of Indigenous Wellbeing – Te Mauri/Pimatisiwin, Journal of Indigenous Research, and Indigenous Knowledge – Other Ways of Knowing. These journals have been selected because they have not been indexed consistently in the biomedical databases. This will ensure comprehensiveness in identifying all available literature and ensuring that scholarly articles written by Indigenous researchers have not been overlooked.

Books and book chapters will be identified in the Scopus and PsycINFO databases and through federated searches in inter-institutional academic library catalogues. Grey literature searches will be carried out using advanced search techniques in Google. Grey literature will be limited to reports or research published by government, academic, and non-profit organizations. Reference lists of key publications will be checked for additional relevant publications, including theses, dissertations, reports, and other articles not retrieved in the online searches. Additional sources known to team members that are potentially eligible for inclusion will be recommended if they are not otherwise captured in the database searches.

Results from the database searches will be imported into Covidence. After duplicate results are removed, two team members will complete the title and abstract screening, then full-text screening for sources that meet our inclusion criteria. We will consider a Cohen’s kappa score of >0.6 acceptable. When two team members disagree about article eligibility, they will meet to discuss their assessment and seek consensus. If consensus cannot be reached, a consistent third reviewer will be consulted to make a final decision. Table 1 describes a detailed version of the inclusion and exclusion criteria. Each included document will be independently appraised and extracted from by any two reviewers on the team. The extracted data will be compared side-by-side to reach consensus.

**Table 1:** Inclusion and exclusion criteria and rationale

Inclusion criteria	Exclusion criteria	Rationale
Printed in English.	Printed in a language other than English.	Nobody on the team is fluent in another language and able to review the documents in the time available.
Reporting on studies that include Indigenous groups or communities.	Reporting on mixed or general populations that may or may not include Indigenous Peoples; reporting on a single person, such as an autoethnography or case study.	We are interested in collective impacts on Indigenous groups, nations, and communities.
Research focuses on Indigenous Peoples within North America, South America, Australia, New Zealand, and Circumpolar Regions	Research that focuses on Indigenous Peoples from places outside of the included areas.	Indigenous Peoples in the listed areas share commonalities in the history of European colonization.
Research reports on mental health risk/protective factors, experiences, outcomes, and/or impacts.	Research that does not explicitly report on mental health and wellness.	The focus of this systematic review is to examine how loss of access to land impacts Indigenous Peoples’ mental health so, it is imperative that the studies included report on this element.
Research is based on land development, extractive industries, and/or contamination leading to loss of access to land - from dams, mines, agriculture and petroleum.	Research that is based on land dispossession from industries other than dams, mines, agriculture, and petroleum.	We are focusing on the four major industrial resource development sectors that result in loss of access to land. We are including contamination that leads to the loss of access to land but not focusing on the biomedical results from contamination unless they are clearly linked to mental health.
Full text is available.	Full text is unavailable.	The full text must be available for appraisal and analysis to be included.
Must be a primary study.	Discussion papers, reviews, or commentaries.	We will exclude all discussion papers, literature reviews, and commentaries for analysis; however, we will read relevant documents as it may inform the background literature and discussion of findings.

### Quality appraisal

For the quality appraisal tool, we will use the Critical Appraisal Skills Programme [33], and include adapted questions so it can be applied to qualitative, quantitative, and mixed research method research designs. We will weigh evidence in the review based on quality. That is, studies that demonstrate a clear connection between land dispossession and mental health (Question10 below) and receive a “yes” to 7 or more of the critical appraisal tool question, will be emphasized in the results. We anticipate that there will be a limited number of studies that meet the inclusion criteria, so we do not plan to exclude studies based on low scores. In addition to changing the questions in the appraisal tool to be inclusive of quantitative and qualitative studies (Question 2 below), we added a relevance question to assess whether a direct connection is made between losing/decreasing access to land and mental health in each study (Question 10 below). The questions in the appraisal tool focus on the quality, rigour, and relevance of the study and include:

1. Was there a clear statement of the aims of the research?
2. Is the methodology appropriate?
3. Was the research design appropriate to address the aims of the research?
4. Was the recruitment strategy appropriate to the aims of the research?
5. Was the data collected in a way that addressed the research issue?
6. Has the relationship between researcher and participants been adequately considered?
7. Have ethical issues been taken into consideration?
8. Was the data analysis sufficiently rigorous?
9. Is there a clear statement of findings?
10. How clear is the connection between losing access to land and mental health made in the study?

### Data extraction

The following information will be extracted from each included study.

1. Reference (full-text citation)
2. Type of document (article, report, other)
3. Geographic setting/location(s)
4. Indigenous Peoples (nations, groups, organizations)
5. Level of Indigenous Peoples involvement in governing research (e.g., initiating the research and identifying priority, guidance and governance on research methodology, gathering data, analysis, how research findings are used, and document writing.)
6. Type of industry (dam, mining, agriculture, or petroleum)
7. Research question(s)
8. Study design/methodology (qualitative, quantitative, mixed methods)
9. Research methods used
10. Tools and other indicators to assess, measure, and document mental health and wellness
11. Reported mental health impacts and outcomes
12. Recommendations

### Data analysis

The extracted data will be exported into an Excel spreadsheet and shared with the full team of authors. First, the demographics and design characteristics of the included studies will be organized by one team member and verified by another. Second, we will hold a series of team meetings to share and discuss the study demographics and study designs; overarching findings, patterns, and relationships between the findings; and identify a sub-group to conduct a thorough and rigorous data analysis. Third, the sub-group of at least four team members will independently identify themes within the extracted field for mental health impacts and outcomes; examine patterns and observations across studies by geography, ways in which land was dispossessed, study methodologies, and the level of Indigenous Peoples’ involvement in the research study; and draft a figure that describes that relationship between land dispossession from industry, the Indigenous Peoples’ mental health and wellbeing, and other arising themes. This sub-group will meet weekly to share, discuss, and re-analyse the data, using an iterative process until everyone in the group is in full agreement about the findings. Having two or more coders is shown to improve rigour, reliability, and accountability [34]. Fourth, the full team will meet to review the preliminary results prepared by the sub-group, provide further input and feedback, and co-develop figures that summarize the reported findings. Fifth, our results will then be shared and discussed with the Community Advisory Team and compared with other literature related to key themes from this review.

## Discussion

This systematic review will assess and synthesize the evidence on the mental health impacts of industrial resource developments that lead to land loss for Indigenous communities. This will include an examination of potential direct and indirect effects. This evidence will be relevant to Indigenous rightsholders and other stakeholders involved in industrial resource development and land use policy, health impact assessments, economic development, and mental health service planning. Potential knowledge users of evidence from this review include Indigenous communities, various levels of government, public health professionals, and industry.

## Strengths and limitations

The primary strength of this review is the collective diverse expertise within our team, which helped to inform the protocol and will aid in data analysis and understanding; this includes having an experienced librarian with expertise in Indigenous health that can develop a comprehensive search strategy [7, 10]. We are focusing the review on four major resource development industries but recognize that other related industries may also report on the mental health impacts of Indigenous populations, which is a potential limitation. Furthermore, by restricting the publication language to English, we may miss relevant papers, particularly from South America, Africa, and Asia. Another anticipated limitation is that most papers will be conceived and written by non-Indigenous authors conducting research about Indigenous Peoples, nations, or communities that they are not part of, which can result in pathologizing Indigenous Peoples and/or missing analyses that reflect local Indigenous knowledge systems.

## Supplementary Information

**Additional file 1.**

**Additional file 2.**

## Data Availability

Not applicable.
