# Supplementary material for: Impact on mental health and wellbeing in Indigenous communities due to land loss resulting from industrial resource development: protocol for a systematic review
Source: Syst Rev. 2022 Jul 20;11:146. doi: 10.1186/s13643-022-02014-2 (PMC9297628; doi:10.1186/s13643-022-02014-2)
Supplement: Supplementary file 2 — Additional file 2. [file 13643_2022_2014_MOESM2_ESM.doc]

**Medline search strategy**

Database: Ovid MEDLINE(R) and Epub Ahead of Print, In-Process & Other Non-Indexed Citations and Daily <1946 to December 31, 2020>

Search Strategy:

--------------------------------------------------------------------------------

1 exp indigenous peoples/ or american native continental ancestry group/ or alaska natives/ or indians, north american/ or inuits/ or Oceanic Ancestry Group/ or Indians, South American/

2 (Indigenous or First Nations or First Nation or Inuit* or metis or Aboriginal or Native American* or alaska native* or (Alaska* adj1 Native*) or Ojibw* or Cree or Athapaskan or Athabaskan or Athabascan or Saulteau* or Wakashan or Dene or Inuk or Tlicho or Haida or Ktunaxa or Tsimshian or Gitsxan or Nisga'a or Haisla or Heiltsuk or Oweenkeno or Kwakwaka'wakw or Nuu chah nulth or Tsilhqot'in or Dakelh or Wet'suwet'en or Sekani or Dunne-za or Dene or Tahltan or Kaska or Tagish or Tutchone or Nuxalk or Salish or Stl'atlimc or Nlaka'pamux or Okanagan or Sec wepmc or Tlingit or Anishinaabe or Anishinabe or Blackfoot or Nakoda or Tasttine or TsuuT'ina or Gwich'in or Tagish or Tutchone or Algonquin or Algonkian or Nipissing or Kahnawake or Mohawk* or Cherokee or Potawatomi or Innu or Maliseet or Mi'kmaq or Passamaquoddy or Haudenosaunee or Cayuga or Lakota or Navajo or Zuni or Hopi or Oneida or Onondaga or Seneca or Tuscarora or Wyandot or Indigeneity or Nunavut* or Iqaluit* or Nunavummiut or Kitikmeot or Kivalliq or qikiqtani or Baffin or Kuujjuaq or Inuvialuit or Nunavik or nunatsiavut or Inupiat or inupiaq or yupik or Yellowknife or northwest territories or Yukon or Whitehorse or Fairbanks or quajigiartiit or eskimo* or maori* or torres strait island* or koori or goori or murri or nyoongah or Nyoongar or Noongar or Nyunga* or koorie or yolngu or Anangu or palawa or nunga or Ngarrindjeri or murray island or mer island or american Indian* or aborigine* or indigen* or Hawaii* or ha waii* or Menominee or Ahousat or Apache tribe or Arapahoe or Bella Coola or Paiute or Shoshone or Blackfeet or Cherokee or Cheyenne or Sioux or Siouan or Choctaw Indian* or Choctaw Nation* or Comanche nation or Kootenai Tribes or Dogrib or Flathead Nation or Flathead Reservation or Havasupai or Pima or Tohono O'odham).mp.

3 (northern Norway or north Norway or Siberia* or canadian north or northern canada or canad* north or nuuk or Svalbard or tromso or finmark or finmarkk or northwest Russia* or khanty or nenets or eskimo* or innu or northern finland or umea or circumpolar or arctic or kalaallit or faroe islands or chukchi or Chukotka* or Greenland* or sami or saami or Aleut* or Iceland* or Reykjavik or northern Sweden or northwest sweden or oulu or Qaujigiartiit).mp.

4 (Amerindian or pueblos originarios or pueblos Indigenas).mp.

5 exp South America/

6 (South America or Argentina or Bolivia or Brazil or Chile or Colombia or Ecuador or "French Guiana" or Guyana or Paraguay or Peru or Suriname or Uruguay or Venezuela or Iberoamerica* or Ibero America* or latin america*).mp.

7 5 or 6

8 (Indian or Indians or Indigenous or Indigen* or Indio or Indios).mp.

9 7 and 8

10 1 or 2 or 3 or 4 or 9

11 exp agriculture/ or exp "conservation of natural resources"/ or forestry/ or industrial development/ or exp "extraction and processing industry"/ or mining/ or coal mining/ or exp "oil and gas industry"/ or exp power plants/

12 exp environmental pollution/ or petroleum pollution/

13 exp environmental pollutants/ or exp hazardous substances/

14 exp Environmental Health/

15 carcinogens/ or exp carcinogens, environmental/

16 exp Environmental Monitoring/

17 droughts/ or floods/

18 exp Environment/

19 11 or 12 or 13 or 14 or 15 or 16 or 17 or 18

20 exp psychological phenomena/ or exp mental disorders/

21 exp self-injurious behavior/ or suicide/ or stress, psychological/

22 20 or 21

23 10 and 19 and 22

24 limit 23 to english language

***************************

**Scopus Search Strategy**

(TITLE-ABS-KEY(Mental* OR psychol* OR psychiatry* OR psychosocial* OR counsel* OR suicid* OR parasuicid* OR addict* OR alcoholism OR alcoholic* OR "alcohol abuse" OR "substance use" OR "substance misuse*" OR "substance abuse*" OR "substance related" OR ptsd OR trauma* OR wellness OR wellbeing OR "well being" OR harm OR harms OR harmful OR harmed OR harming OR grief OR emotion* OR resilien* OR anxiety)) AND (((TITLE-ABS-KEY(Mines OR mining OR mine OR dam OR dams OR flood* OR extractivism OR hydroelectric* OR "hydro electric*" OR "hydro power" OR hydropower OR "power plant*" ) OR TITLE-ABS-KEY(forest* OR deforest* OR petrochemical* OR petroleum OR agricultur* OR "industrial development*" OR pipeline* OR "energy industry" OR "renewable energy" OR "energy sector" OR "energy development" ) OR TITLE-ABS-KEY("oil spill*" OR deforestation OR logging OR clearcut* OR "clear cut*" OR forestry OR "water rights" OR "oil reserves" OR "oil sands" OR frack* OR "hydraulic fracturing" OR "natural gas" OR "sour gas" OR drilling) OR TITLE-ABS-KEY(methylmercury OR mercury OR uranium OR "lead poisoning" OR arsenic OR cyanide OR cadmium OR contamin* OR expropriat* OR "environmental health" OR "environmental impact*" OR "environmental justice" OR pollut* ) OR TITLE-ABS-KEY("environmental degradation" OR "environmental legislation" OR "environmental law*" OR "environmental toxi*" OR carcinogen* OR pcb* OR erosion OR "water security" OR "water insecurity") OR TITLE-ABS-KEY("environmental management" OR "environmental planning" OR "environmental issue*" OR "environmental exposure*" ))) OR ((TITLE-ABS-KEY(Technological W/2 hazard* ) OR TITLE-ABS-KEY(technological W/2 disaster*) OR TITLE-ABS-KEY(oil W/2 gas) OR TITLE-ABS-KEY(extract* W/2 industr*) OR TITLE-ABS-KEY(land W/2 develop*) OR TITLE-ABS-KEY(Extract* W/2 resource*) OR TITLE-ABS-KEY(loss W/2 land)))) AND (((TITLE-ABS-KEY(Indigenous OR "First Nations" OR Inuit* OR Metis OR Aboriginal OR "Native American" OR "Native Americans" OR "Alaska Native" OR "Alaskan Native" OR "Alaska natives" OR "Alaskan natives" OR "Native Alaskans") OR TITLE-ABS-KEY("Indians north American" OR "American native continental ancestry group" OR "oceanic ancestry group" OR aborigine* OR indigen* OR Hawaii* OR "Ha waii*" )OR TITLE-ABS-KEY(Ojibw* or Cree or Athapaskan or Athabaskan or Athabascan or Saulteau* or Wakashan or Dene or Inuk or Tlicho or Haida or Ktunaxa or Tsimshian or Gitsxan or Nisga'a or Haisla or Heiltsuk or Oweenkeno ))) OR ((TITLE-ABS-KEY( Kwakwaka'wakw or Nuu chah nulth or Tsilhqot'in ) OR TITLE-ABS-KEY(Dakelh or Wet'suwet'en or Sekani ) OR TITLE-ABS-KEY(Dunne-za or Dene or Tahltan or Kaska or Tagish or Tutchone or Nuxalk or Salish or Stl'atlimc or Nlaka'pamux or Okanagan or "Sec wepmc" or Tlingit ) OR TITLE-ABS-KEY( Anishinaabe or Anishinabe or Blackfoot or Nakoda or Tasttine or TsuuT'ina or Gwich'in or Tagish or Tutchone or Algonquin or Algonkian or Nipissing ) OR TITLE-ABS-KEY( Kahnawake or Mohawk* or Cherokee or Potawatomi or Maliseet or Mi'kmaq or Passamaquoddy or Haudenosaunee ))) OR ((TITLE-ABS-KEY(Lakota or Navajo or Zuni or Hopi or Oneida or Onondaga or Tuscarora or Wyandot or Indigeneity) OR TITLE-ABS-KEY(maori* or "torres strait islander" OR "torres strait islanders" or koori or goori or murri or nyoongah or Nyoongar or Noongar or Nyunga* or koorie or yolngu ) OR TITLE-ABS-KEY(Anangu or palawa or nunga or Ngarrindjeri or "murray island" or "mer island" or "american Indian" OR "American Indians" or aborigine* or indigen* ) OR TITLE-ABS-KEY(Menominee or Ahousat or "Apache tribe" or Arapahoe or "Bella Coola" or Paiute or Shoshone or Blackfeet or Cherokee or Cheyenne or Sioux or Siouan ) OR TITLE-ABS-KEY("Choctaw Indian*" or "Choctaw Nation*" or "Comanche nation" or "Kootenai Tribe*" or Dogrib or "Flathead Nation" or "Flathead Reservation" or Havasupai or Pima ) OR TITLE-ABS-KEY(Tohono O'odham))) OR ((TITLE-ABS-KEY("northern Norway" OR "north Norway" OR Siberia* OR "canadian north" OR "northern canada" OR "canada's north" OR nuuk OR Svalbard OR tromso OR finmark OR finmarkk OR "northwest Russia*" OR khanty OR nenets) OR TITLE-ABS-KEY(eskimo* OR innu OR "northern finland" OR umea OR circumpolar OR arctic OR Nunavut* OR Iqaluit OR Nunavummiut OR Kitikmeot OR Kivalliq OR Qikiqtani OR Baffin OR kuujjuaq OR Inuvialuit OR Nunavik OR Nunatsiavut OR Inupiat OR Inupiaq OR yupik) OR TITLE-ABS-KEY(kalaallit OR "faroe islands" OR chukchi OR Chukotka* OR inuit* OR Inuk OR "Alaskan native" OR "alaska native" OR "native alaskan" OR "native alaskans" OR Yup'ik OR yellowknife OR "northwest territories" OR Yukon OR Whitehorse) OR TITLE-ABS-KEY( Fairbanks OR Greenland* OR sami OR saami OR Aleut* OR Iceland* OR Reykjavik OR "northern Sweden" OR "northwest sweden" OR oulu OR Qaujigiartiit))) OR ((TITLE-ABS-KEY(indigenous OR "native american*" OR aboriginal OR indian) AND TITLE-ABS-KEY(dakota))) OR ((TITLE-ABS-KEY("South America" OR Argentina OR Bolivia OR Brazil OR Chile OR Colombia OR Ecuador OR "French Guiana" OR Guyana OR Paraguay OR Peru OR Suriname OR Uruguay OR Venezuela OR Iberoamerica* OR "Ibero America*" OR "latin america*") AND TITLE-ABS-KEY(Indian OR Indians OR Indigenous OR Indigen* OR Indio OR Indios))) OR (TITLE-ABS-KEY(Amerindian OR "pueblos originarios" OR "pueblos Indigenas" ))) AND ( LIMIT-TO ( DOCTYPE,"ar" ) OR LIMIT-TO ( DOCTYPE,"re" ) ) AND ( LIMIT-TO ( LANGUAGE,"English" ) )

**Sources consulted [JL, NP] for search terms for Indigenous People:**

Appropriate Terminology, Representations, and Protocols of Acknowledgement for Aboriginal and Torres Strait Islander Peoples. [Internet]. Bedford Park, SA.: Flinders University; 2012. Available from: [https://dlb.sa.edu.au/tlsmoodle/pluginfile.php/14777/mod_folder/content/0/2_%20Appropriate%20Terminology%2C%20Indigenous%20Australians.pdf?forcedownload=1](https://dlb.sa.edu.au/tlsmoodle/pluginfile.php/14777/mod_folder/content/0/2_ Appropriate Terminology%2C Indigenous Australians.pdf?forcedownload=1)

Campbell, S, Dorgan, M, Tjosvold, L. Rev. March 8, 2016. [Internet]. [Edmonton]: John W. Scott Health Sciences Library, University of Alberta. Available from: <https://guides.library.ualberta.ca/health-sciences-search-filters/indigenous-peoples>

King, J, Masotti, P, Dennem, J, Hadani, S, Linton, J, Lockhart, B, et al. The Culture Is Prevention Project: Adapting the Cultural Connectedness Scale for Multi-Tribal Communities. Am Indian Alsk Native Ment Health Res. 2019;26(3):104‐135. doi:10.5820/aian.2603.2019.104

Lowitja Institute. LIt.Search [Internet]. Carlton South, Vic.: The Lowitja Institute; 2020. Available from: <https://www.lowitja.org.au/page/research/lit-search>

Native Health Database [Internet]. Albuquerque, NM: University of New Mexico, [nd]. Available from: <https://hslic-nhd.health.unm.edu/>

Pollock, N. J., Naicker, K., Loro, A., Mulay, S., & Colman, I. Global incidence of suicide among Indigenous peoples: a systematic review. BMC Medicine. 2018; *16*(1), 145.
